# Early inflammatory signal in late-diagnosed anastomotic leakage after anterior resection

**DOI:** 10.1093/bjsopen/zrag127

**Published:** 2026-09-29

**Authors:** Martin Rutegård, Josefin Segelman, Peter Matthiessen, Marie-Louise Lydrup, Jennifer Park, the RectoLeak Study Group, A Gerdin, O Sjöström, M Staffan, S Jangmalm, H Royson, K Tsimogiannis, K Anderin, J Nygren, J Hurtig, Parisa Golshani

**Affiliations:** Department of Surgery, SSORG—Scandinavian Surgical Outcomes Research Group, Institute of Clinical Sciences, Sahlgrenska Academy, University of Gothenburg, Gothenburg, Sweden; Department of Surgery, Sahlgrenska University Hospital/Östra, Gothenburg, Sweden; Department of Molecular Medicine and Surgery, Karolinska Institutet, Stockholm, Sweden; Department of Surgery, Ersta Hospital, Stockholm, Sweden; Department of Surgery, Faculty of Medicine and Health, Örebro University, Örebro, Sweden; Department of Surgery, Skåne University Hospital, Malmö, Lund University, Lund, Sweden; Department of Surgery, SSORG—Scandinavian Surgical Outcomes Research Group, Institute of Clinical Sciences, Sahlgrenska Academy, University of Gothenburg, Gothenburg, Sweden; Department of Surgery, Sahlgrenska University Hospital/Östra, Gothenburg, Sweden

**Keywords:** rectal cancer, inflammation, anastomotic leak, detection

A substantial proportion of anastomotic leakage after anterior resection for rectal cancer is diagnosed late in the postoperative period^1^. It is often assumed that, in reality, these leaks occur earlier; in particular, the use of a defunctioning stoma may reduce symptoms to such an extent that leaks escape clinical detection^2^. This study investigated this assumption by assessing elevations in C-reactive protein (CRP) levels.

This is a post hoc exploratory analysis. Data were used from a multicentre national retrospective cohort study including patients who underwent anterior resection for rectal cancer at 11 centres in Sweden between 2014 and 2018 with 1-year follow-up concerning anastomotic leakage^3^. The study was approved by the Regional Board of the Ethical Committee in Uppsala, Sweden (DNR: 2020-02285, 2020-03795, 2020-05126).

Anastomotic leakage was defined and classified according to the International Study Group of Rectal Cancer^4^. Exposure in the present study was the diagnosis of an early (≤ 14 days) or late (> 14 days) leak, whereas outcome was the maximum CRP level within the first 14 days after surgery. Linear regression with adjustment for confounding was performed, considering age, sex, body mass index, American Society of Anesthesiologists grade, clinical tumour stage, neoadjuvant therapy, total or partial mesorectal excision, splenic flexure mobilization, ligation level of the inferior mesenteric artery, minimally invasive surgery, intraoperative bleeding, operation time, and defunctioning stoma (*Table S1*).

After exclusion of eight patients with missing CRP values, 1118 patients were analysed. Of these patients, 109 (9.8%) had a leakage diagnosed within 14 days, whereas 122 (10.9%) were diagnosed later. Early and late leaks were detected on median postoperative day 7 (interquartile range (i.q.r.) 5–10) and postoperative day 39 (i.q.r. 21–95), respectively. There were 3 grade A (2.8%), 66 grade B (60.6%), and 40 grade C (36.7%) leaks in the early group, compared with 31 (25.4%), 64 (52.5%), and 27 (22.1%) grade A, B, and C leaks, respectively, in the late group (*Table S2*).

As shown in *Fig. 1*, the median maximum CRP was 108.5 (i.q.r. 61–169) mg/L in patients without leakage, compared with 270 (i.q.r. 200–370) and 167.5 (i.q.r. 120–237) mg/L in patients with early and late leakage, respectively (Kruskal–Wallis *P* < 0.001). Compared with patients without leakage, the adjusted increase in CRP was 132 (95% confidence interval (c.i.) 113–152) mg/L for early leaks and 42 (95% c.i. 24–60) mg/L for late leaks. Sensitivity analysis using 30 days as the cut-off for early *versus* late leakage confirmed the findings (*Fig. S1*), and restriction to the 789 patients with a defunctioning stoma yielded similar results (*Fig. S2*).

**Figure 1:**
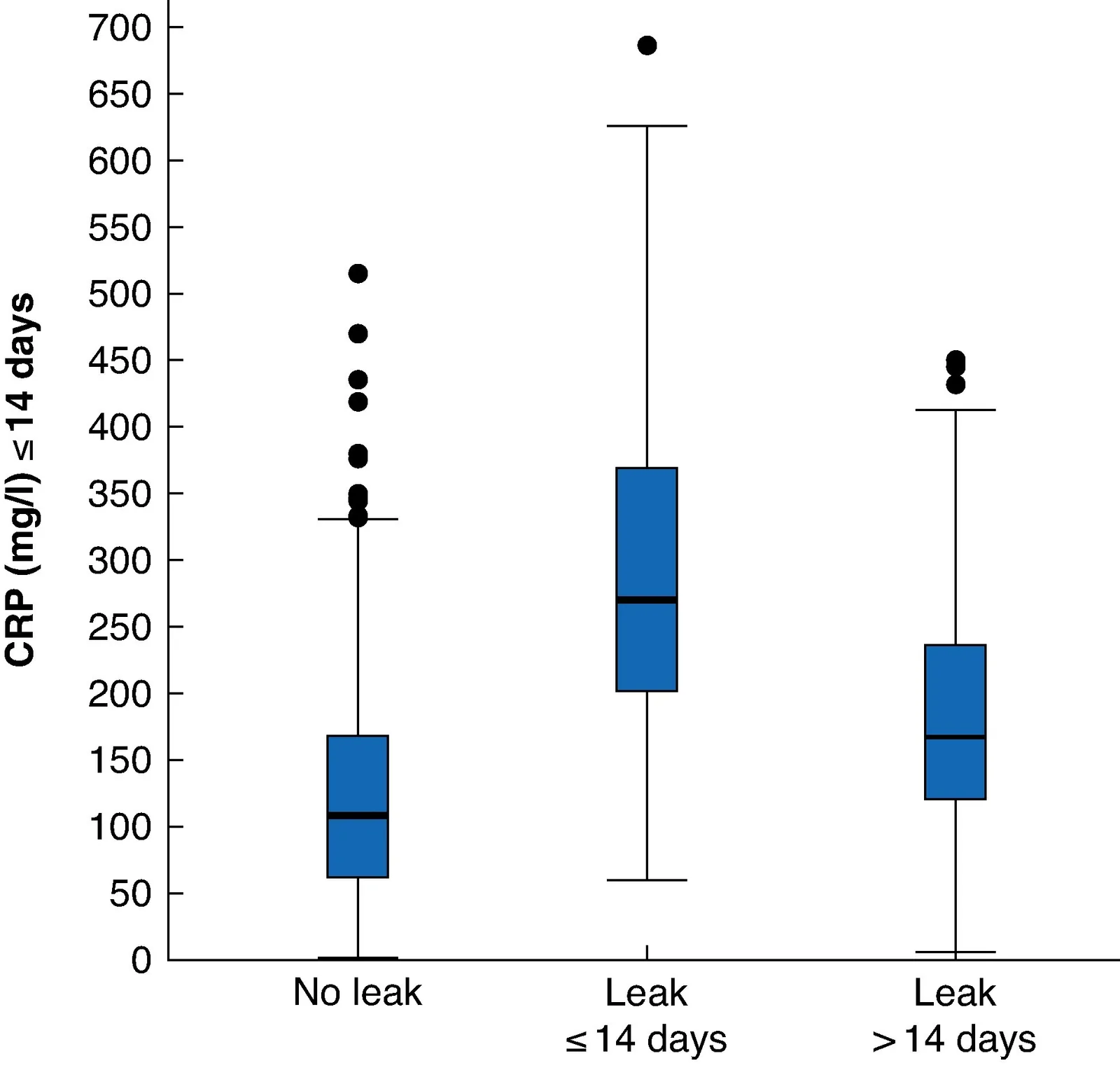
Maximum postoperative CRP level (mg/L) within 14 days after surgery according to the timing of anastomotic leakage after anterior resection for rectal cancer (*n* = 1118) Horizontal lines indicate median CRP levels; boxes indicate the interquartile range, whiskers indicate the range, and symbols indicate outliers. CRP, C-reactive protein.

Maximum CRP within the first 2 weeks after surgery was markedly higher in patients with an anastomotic leakage occurring later than in patients without any leakage. These findings support the hypothesis that some late-diagnosed leaks may have an early postoperative biological signal and may therefore represent leaks that were present, but not clinically detected, earlier in the postoperative course. This may alleviate concerns of inducing immortality bias when evaluating effects of anastomotic leakage on long-term outcomes^5^; if leaks actually occur close to the time of surgery, even when diagnosed later, there is no need to assume that the starting point (‘time zero’) differs from the day of the operation, which is typically used in such analyses. A more clinically oriented implication may be that systematic imaging, endoscopy, or other investigations are warranted with an unexplained increase in CRP even in otherwise well patients, enabling earlier detection and treatment.

Limitations of this study include the retrospective design with different diagnostic modalities and algorithms in each study centre, as well as the non-standardized CRP collection. To assess the true leakage incidence, real-time monitoring would likely be necessary, although this may never be realized. Despite extensive adjustment for covariates reflecting demographics, tumour extent, preoperative treatment, and operative complexity, the available data may not have adequately captured these, resulting in residual confounding, acting as an alternative explanation to the findings.

## Collaborators

Members of the RectoLeak Study Group are: A. Gerdin, O. Sjöström (Umeå University, Umeå, Sweden); M. Staffan (Falun Hospital, Falun, Sweden); S. Jangmalm, H. Royson (Växjö Hospital, Växjö, Sweden); K. Tsimogiannis (Uppsala University, Uppsala, Sweden); K. Anderin, J. Nygren (Ersta Hospital and Karolinska Institutet (KIDS), Stockholm, Sweden); J. Hurtig (Sunderby Hospital, Luleå, Sweden); Parisa Golshani (Danderyd Hospital, Danderyd, Sweden)

## Supplementary Material

zrag127_Supplementary_Data

## Data Availability

Data will be made available upon reasonable request to the corresponding author.

## References

[1] Borstlap WAA, Westerduin E, Aukema TS, Bemelman WA, Tanis PJ; Dutch Snapshot Research Group. Anastomotic leakage and chronic Presacral Sinus formation after low anterior resection: results from a large cross-sectional study. Ann Surg 2017;266:870–877 10.1097/SLA.0000000000002429 28746154

[2] Emile SH, Khan SM, Garoufalia Z, Silva-Alvarenga E, Gefen R, Horesh N et al When is a diverting stoma indicated after low anterior resection? A meta-analysis of randomized trials and meta-regression of the risk factors of leakage and complications in non-diverted patients. J Gastrointest Surg 2022;26:2368–2379 10.1007/s11605-022-05427-5 35915378

[3] Rutegard M, Svensson J, Segelman J, Matthiessen P, Lydrup ML, Park JM et al Anastomotic leakage in relation to type of mesorectal excision and defunctioning stoma use in anterior resection for rectal cancer. Dis Colon Rectum 2024;67:398–405 10.1097/DCR.0000000000003050 37994449

[4] Rahbari NN, Weitz J, Hohenberger W, Heald RJ, Moran B, Ulrich A et al Definition and grading of anastomotic leakage following anterior resection of the rectum: a proposal by the International Study Group of Rectal Cancer. Surgery 2010;147:339–351 10.1016/j.surg.2009.10.012 20004450

[5] Denost Q, Rouanet P, Faucheron JL, Panis Y, Meunier B, Cotte E et al Impact of early biochemical diagnosis of anastomotic leakage after rectal cancer surgery: long-term results from GRECCAR 5 trial. Br J Surg 2021;108:605–608 10.1093/bjs/znab003 33793764

