# Supplementary material for: Early inflammatory signal in late-diagnosed anastomotic leakage after anterior resection
Source: BJS Open. 2026 Sep 29;10(5):zrag127. doi: 10.1093/bjsopen/zrag127 (PMC13623004; doi:10.1093/bjsopen/zrag127)
Supplement: Supplementary file 1 [file zrag127_supplementary_data.docx]

## Supplementary Material for the article “Early inflammatory signal in late-diagnosed anastomotic leakage after anterior resection”

### Authors

Martin Rutegård (1, 2), Josefin Segelman (3), Peter Matthiessen (4), Marie-Louise Lydrup (5), Jennifer Park (1, 2); on behalf of the RectoLeak study group*.

### Affiliations

1) Department of Surgery, SSORG – Scandinavian Surgical Outcomes Research Group, Institute of Clinical Sciences, Sahlgrenska Academy, University of Gothenburg, Gothenburg, Sweden.

2) Department of Surgery, Sahlgrenska University Hospital/Östra, Region Västra Götaland, Gothenburg, Sweden.

3) Department of Molecular Medicine and Surgery, Karolinska Institutet, and Department of Surgery, Ersta Hospital, Stockholm, Sweden.

4) Department of Surgery, Faculty of Medicine and Health, Örebro University, Örebro, Sweden.

5) Department of Surgery, Skåne University Hospital, Malmö, Lund University, Lund, Sweden.

### Corresponding author:

Martin Rutegård (martin.rutegard@gu.se)

Department of Surgery, Institute of Clinical Sciences, Sahlgrenska Academy, University of Gothenburg

SE-901 85 Gothenburg, Sweden

ORCID: 0000-0002-0974-6373

Twitter: @martin_rutegard

### Supplementary Materials - Index

| **Supplementary Figures and Tables** |  |
| --- | --- |
| Table S1 | *page 2* |
| Table S2 | *page 3* |
| Figure S1 | *page 4* |
| Figure S2 | *page 4* |
|  |  |

### Supplementary Figures and Tables

**Table S1.** Covariates used in linear regression model.

| Covariate | Categories |
| --- | --- |
| Sex | Male, female |
| Age (years) | Continuous |
| Body mass index (kg/m^2^) | Continuous |
| American Society of Anesthesiologists’ classification | I, II, III–IV |
| Clinical T stage | cT1–2, cT3, cT4, cTX |
| Neoadjuvant treatment | None, radiotherapy only, chemoradiotherapy |
| Type of mesorectal excision | Partial, total |
| Splenic flexure mobilisation | No, yes |
| High tie of inferior mesenteric artery | No, yes |
| Surgical approach | Open/converted, laparoscopic, robotic |
| Intraoperative bleeding (ml) | Continuous |
| Operative time (ml) | Continuous |
| Defunctioning stoma | No, yes |

**Table S2.** Demographics, tumour stage, neoadjuvant and operative treatment characteristics of 1,118 patients operated for rectal cancer at 11 centres in Sweden 2014–2018, by anastomotic leak timing.

|  | No leak | Leak day ≤14 | Leak day >14 |
| --- | --- | --- | --- |
|  | **N=884** | **N=109** | **N=122** |
| Age (years) | 67.0 (60.0-73.0) | 66.0 (59.0-72.0) | 66.0 (59.0-73.0) |
| Sex |  |  |  |
| Male | 517 (58.5%) | 75 (68.8%) | 88 (72.1%) |
| Female | 367 (41.5%) | 34 (31.2%) | 34 (27.9%) |
| Body mass index (kg/m^2^) | 25.5 (23.2-28.3) | 25.8 (23.1-29.0) | 26.0 (24.1-29.1) |
| ASA fitness grade |  |  |  |
| I | 193 (21.8%) | 22 (20.2%) | 27 (22.1%) |
| II | 536 (60.6%) | 64 (58.7%) | 67 (54.9%) |
| III–V | 148 (16.7%) | 21 (19.3%) | 27 (22.1%) |
| Missing | 7 (0.8%) | 2 (1.8%) | 1 (0.8%) |
| Clinical T stage |  |  |  |
| cT1–2 | 310 (35.1%) | 33 (30.3%) | 38 (31.1%) |
| cT3 | 459 (51.9%) | 65 (59.6%) | 63 (51.6%) |
| cT4 | 90 (10.2%) | 10 (9.2%) | 19 (15.6%) |
| cTX | 23 (2.6%) | 1 (0.9%) | 2 (1.6%) |
| Missing | 2 (0.2%) | 0 (0.0%) | 0 (0.0%) |
| Neoadjuvant therapy |  |  |  |
| None | 476 (53.8%) | 39 (35.8%) | 36 (29.5%) |
| Radiotherapy only | 269 (30.4%) | 52 (47.7%) | 53 (43.4%) |
| Chemoradiotherapy | 139 (15.7%) | 18 (16.5%) | 33 (27.0%) |
| Type of mesorectal excision |  |  |  |
| Total | 662 (74.9%) | 98 (89.9%) | 113 (92.6%) |
| Partial | 222 (25.1%) | 11 (10.1%) | 9 (7.4%) |
| Splenic flexure mobilization |  |  |  |
| No | 531 (60.1%) | 55 (50.5%) | 67 (54.9%) |
| Yes | 297 (33.6%) | 43 (39.4%) | 38 (31.1%) |
| Missing | 56 (6.3%) | 11 (10.1%) | 17 (13.9%) |
| High tie of inferior mesenteric artery |  |  |  |
| No | 530 (60.0%) | 62 (56.9%) | 83 (68.0%) |
| Yes | 339 (38.3%) | 45 (41.3%) | 37 (30.3%) |
| Missing | 15 (1.7%) | 2 (1.8%) | 2 (1.6%) |
| Surgical approach |  |  |  |
| Open/converted | 467 (52.8%) | 62 (56.9%) | 71 (58.2%) |
| Laparoscopy | 165 (18.7%) | 22 (20.2%) | 14 (11.5%) |
| Robotic | 246 (27.8%) | 24 (22.0%) | 37 (30.3%) |
| Missing | 6 (0.7%) | 1 (0.9%) | 0 (0.0%) |
| Intraoperative bleeding (ml) | 150 (50–400) | 200 (100–500) | 300 (100–500) |
| Operative time (min) | 252 (189–349) | 292 (199–397) | 270 (207–349) |
| Defunctioning stoma |  |  |  |
| No | 184 (20.8%) | 21 (19.3%) | 7 (5.7%) |
| Yes | 700 (79.2%) | 88 (80.7%) | 115 (94.3%) |

Data are presented as median (interquartile range) for continuous measures, and n (%) for categorical measures. ASA = American Society of Anesthesiologists’.

**Figure S1**. Maximum postoperative C-reactive protein (CRP) level (mg/L) within 14 days by timing of anastomotic leakage after anterior resection for rectal cancer (n=1,118), using cut-off 30 days for early vs late leak. Median levels with interquartile ranges and outliers.

**Figure S2.** Maximum postoperative C-reactive protein (CRP) level (mg/L) within 14 days by timing of anastomotic leakage, with restriction to defunctioned patients after anterior resection for rectal cancer (n=789). Median levels with interquartile ranges and outliers.
